# E-B-ocimene and brood cannibalism: Interplay between a honey bee larval pheromone and brood regulation in summer dearth colonies

**DOI:** 10.1371/journal.pone.0317668

**Published:** 2025-02-06

**Authors:** Mark J. Carroll, Nicholas Brown, Eden Huang

**Affiliations:** Carl Hayden Bee Research Center USDA-ARS, Tucson, Arizona, United States of America; University of Alberta, CANADA

## Abstract

Honey bees balance colony populations against available food resources by adjusting brood rearing during nutritionally-stressed periods. Workers limit colony populations primarily through brood cannibalism of eggs and young larvae but often resume brood rearing when conditions improve. However, extended brood cannibalism reduces brood and removes brood signals that mediate brood rearing, such as E-β-ocimene, a volatile pheromone produced by eggs, young larvae, prepupae and ovipositing queens. We examined the effects of pollen supplementation on ocimene signaling in nutritionally-stressed colonies. Pollen-deprived colonies showed declines in ocimene emissions that coincided with sustained brood cannibalism of pheromone-producing brood. In contrast, pollen-supplemented colonies reared more brood and released more ocimene. Twelve day old workers that completed adult development in pollen-deprived colonies had less well developed hypopharyngeal glands and fat bodies than workers that matured in pollen-supplemented colonies. Given that ocimene emissions increased once brood rearing resumed, we considered the possibility that ocimene may help suppress brood cannibalism and support egg retention in nutritionally stressed nuc colonies. Broodless nucleus frames were treated with synthetic ocimene releases equivalent to 3,744 L2-L3 larvae. All ocimene-supplemented nucs retained large numbers of eggs and young larvae four days after initial treatment. By contrast, half of the unsupplemented nucs cannibalized all of their eggs and L1 larvae. Most of the remaining unsupplemented nuc colonies retained fewer eggs and L1 larvae than ocimene supplemented nuc colonies. E-B-ocimene may prime nutritionally stressed workers to increase brood rearing during dearth periods by projecting the presence of healthy eggs and young larvae.

## Introduction

Social insects with perennial colonies balance their populations in response to fluctuations in available resources. Western honey bees *Apis mellifera* L. maintain colonies year round on floral resources, a seasonally variable and sometimes unpredictable food resource [1,2]. Bees feeds on nectar as their primary carbohydrate source and pollen as their primary source of lipids, sterols, and protein [3–6]. Both floral rewards are nutritionally rich but seasonally ephemeral and prone to erratic cycles of abundance and scarcity. As a highly eusocial species, honey bees overcome many of the disadvantages of floral nutrition through social coordination and context-dependent task responses. Most notably, the base mechanisms of food collection, storage, distribution, and allocation are not controlled directly by most food consumers. Rather, food is preserved and fed to many consumers (i.e., larvae, queens) by the adult workers themselves [3]. Workers are capable of communicating the presence and location of a broad array of floral species for mass foraging [6,7]. Workers maintain collected nectar and pollen in preserved storage forms that last for weeks or months before consumption. In addition, nest workers control nutrient distribution to colony dependents through mass provisioning of glandular secretions (young larvae), piecemeal feeding (older worker larvae), and adult-to-adult trophallactic exchanges (young adult workers, queens, and drones) [8–14].

Workers routinely balance colony populations against nutritional shortfalls by mechanisms that reduce the colony population to sustainable levels. Workers respond to declining food availability by reducing brood care and engaging in brood cannibalism of eggs and young larvae [15–21]. Workers preferentially neglect younger larvae more than older larvae and consume poorly nursed younger larvae first [16,18]. Brood cannibalism during stressed periods is most pronounced in older eggs and younger (L1-L3) larvae. These life stages are normally mass provisioned with larval jelly, which is the first major worker nutritional investment that workers give to larvae [17]. Notably, older larvae are usually fed sufficiently, and pupae and adults are rarely cannibalized for nutrients [15]. Cannibalism of young life stages reduces the colony’s consumer population, is thought to improve care and quality of surviving dependent colony members by freeing up resources, and recycles nutrients back into the colony [16,18,20,22]. Nutritionally stressed workers also reduce adult-to-adult trophallaxis and engage in early capping of 5^th^ instar larvae [16,19]. Collectively, nutritionally-stressed colonies direct limited nutrient resources to support older larval development (i.e., favor worker quality over quantity by reducing brood populations to more sustainable numbers) and preserve worker competencies until conditions improve [22]. Workers also adjust foraging effort in response to forage and food availability [23,24]. In particular, signs of increased forage availability such as dilute nectar or fresh pollen may induce workers to forage [25]. Conversely, workers may decrease foraging effort if pollen stores are abundant [26].

These colony nutritional balancing mechanisms can become severely strained during prolonged dearth, when there is insufficient forage to replace food consumed by the colony over long periods of time. Honey bee colonies experience chronic forage shortages due to natural dearth, drought, freezes, and poor weather [2,17,18,27,28]. During extended dearth, bees often deplete colony pollen stores and become completely dependent on internal nutrient stores within worker bodies [3,29,30]. Chronic malnutrition may also impact worker functional lifespan and task capabilities [3,9,20,31–33]. Adult workers that develop under prolonged nutritional stress may exhibit stunted growth, underdeveloped tissues, and engage in precocious foraging [20,34,35]. Critically, nursing capabilities of severely malnourished workers can be compromised by underdeveloped HPG and depleted internal nutrient stores [36–40]. While flexible, full nursing capacities cannot always be easily restored once forage becomes available, and atrophied HPG rarely fully redevelop, even in the presence of fresh abundant food [41–43]. Poorly functioning hypopharyngeal glands can also form a bottleneck in future brood rearing capabilities given that young larvae completely depend on larval jelly for food [9,44]. Eventually, prolonged brood cannibalism can impact the colony both by reducing the stock of replacement workers and decimating brood populations [22]. For these reasons, brood rearing is often precarious in nutritionally-stressed colonies that have fewer workers and nutrient reserves than well provisioned colonies, given the metabolic and longevity costs of rearing brood to workers [31,45,46]. Despite these challenges, nutritionally-stressed colonies often manage to resume brood rearing once forage becomes available again.

One system that is likely impacted by colony responses to poor nutrition is pheromone communication that mediates brood care and worker task functions. Workers maintain flexibility in meeting colony needs in part through age-related polyethism [7,47]. As adults, honey bee workers tend to perform different tasks as they mature through a series of temporal castes from nest activities to foraging. Conversely, dependent colony members such as eggs, larvae, pupae, queens, and drones communicate their needs to workers both directly through individual interactions and also by mass signaling at the colony level [48]. Bees of different castes and life stages produce pheromones that alter worker interactions with other bees both through immediate releaser effects and long term primer effects on worker maturation and physiology [see 49–51 for reviews]. One such pheromone is the terpene E-β-ocimene, a volatile emitted at high levels by young larvae, prepupae and ovipositing queens that has been recently detected from eggs [48,52–56]. Ocimene is released by many life stages that need minimal worker care (eggs, young larvae, prepupae). Ocimene causes adult workers to mature faster to foragers, promotes growth of jelly-producing hypopharyngeal glands (HPG), and modestly increases pollen foraging effort (less than brood itself) [38,48,56–59]. Ocimene is also known to directly mediate brood care responses by increasing worker visits to starving younger and older larvae [60]. Ocimene works in tandem with other colony regulatory pheromones (brood ester pheromone (BEP) from older larvae, queen mandibular pheromone (QMP) from queens, and ethyl oleate (EO) from foragers) to structure worker maturation to the needs of different colony members [24,48,61–63]. Worker exposure to ocimene occurs both at the individual interaction and colony scale and is likely dependent on the number of pheromone-producing brood present [48,59]. However, nutritionally-stressed colonies that decrease brood rearing may experience reductions in brood produced pheromones, with unknown effects on brood care.

In this study, we asked whether E-β-ocimene affects brood care in its most fundamental aspect, namely, if ocimene affects whether younger immatures are cannibalized or not. We explored the interplay between colony brood regulation mechanisms, a brood produced pheromone, and resumption of brood rearing in nutritionally-stressed colonies. We focused on the response of drought-stressed colonies to a short term pollen increase, an event known to have stimulatory effects on brood rearing in nutritionally challenged bees. Here, we examined how increased pollen availability changes colony production of E-β-ocimene given its association with eggs, young larvae, and ovipositing queens. E-β-ocimene is a highly volatile compound that can be readily monitored at either the frame or whole colony level [53], and can be reliably supplemented in the nest environment using capillary vial release mechanisms [see 64 for original method developed with wasps]. We also asked whether synthetically applied ocimene itself, by simulating the presence of eggs and young larvae, could stimulate dearth colonies to reduce egg cannibalism and increase brood rearing [48,52,53,56]. Could E-β-ocimene in this context function as a signal to stressed colony workers to reduce brood culling?

## Materials and methods

### Establishment of colonies and nucleus colonies

Colonies were established in separate years for the 2011 pollen supplementation experiment (Experiment 1) and the 2024 ocimene supplementation experiment (Experiment 2) under different degrees of forage deprivation (severe drought in 2011; seasonal summer dearth in 2024). Both sets of colonies were established at Carl Hayden Bee Research Center (CHBRC) in Tucson, Arizona. This apiary is located at the edge of a mesquite-palo verde riparian woodland surrounded by urban development and agricultural fields [2].

For the 2011 pollen supplementation experiment (Experiment 1), honey bees and commercial queens derived from Italian bees were established in mid-April as 3 lb. (1.36 kg) packages (~13,000 to 14,000 workers) in Langstroth deep hive equipment. Colonies were fed dilute sucrose sugar syrup (2:1 v/v) and pollen patty (9:1 sucrose:pollen) containing trap-collected corbicular pollen of mixed origin (Sonoran Desert Mixture, Leonard Hines, Wilcox, AZ, USA) *ad libetum*. Local Sonoran desert colonies usually experience a bimodal pattern of pollen and nectar availability with peak forage availability in late spring/early summer and late summer/early fall [2]. However, forage was severely impacted by two hard freezes in February 2011 and a severe extended drought from September 2010 to December 2011. Colonies initially foraged on palo verde (*Cercidium microphyllum*), *Acacia* spp. and creosote (*Lattea divaricata*) for three weeks in April 2011. However, forage was severely reduced during the remainder of the spring, summer, and early fall of 2011. The mesquite (*Prosopis spp.*) flow from late April to mid June, which is the main annual floral source of nectar and pollen, almost completely failed. Local field colonies were almost completely dependent on periodic supplementation with pollen patty and dilute sucrose sugar syrup (2:1 v/v) from October 2010 through December 2011 for survival. By early August 2011, adult workers were severely malnourished (average nest worker fresh mass without gut contents ( ± SE) of 87.2 ±  2.7 mg)) compared to workers from non-drought years (97.2 ±  3.2 mg)). Despite severe chronic nutritional stress, the field colonies reared sufficient amounts of brood to maintain colony populations.

For the 2024 ocimene supplementation experiment (Experiment 2), nucleus colonies (nucs) were made from colonies headed by commercial queens during a non-drought year. Source colonies were overwintered locally or transferred from almond pollination. Local colonies experienced above average nectar and pollen forage from March to June 2024 and dearth from late June to mid-September. Nucs were established as splits in June and July 2024 with a commercial queen derived from Italian bees, 3,200-5,000 workers, one Langstroth deep brood frame, two honey frames and one empty cell frame. Nucs were placed under shade cloth structures near a water source to mitigate heat stress. Nucs were equalized twice and fed 200 g Global Patties (25% pollen custom formulation, Global Patties, Inc., Airdrie, AB, Canada) every two weeks during the late summer dearth (from early August to mid-September). Local summer dearth colonies have adequate honey stores and access to minor pollen availability but limited nectar flows. As such, brood populations were reduced by 64% to 86% over the dearth.

### Pollen supplementation and deprivation of drought-stressed arena colonies

In 2011, drought-stressed field colonies were isolated in small flight arenas to examine the effects of short-term pollen supplementation on ocimene signaling in chronically malnourished colonies (S1 Fig). Each flight arena consisted of a quad subdivision (0.9 m W x 1.5 m L x 1.8 m H) of a hoop frame cold house (1.8 m W x 3.1 m L x 1.8 m H) covered by fine nylon mesh (Growers Supply, Inc., Dyersville, IA, USA). Colonies experienced better survivorship in these small flight arenas than in larger arenas and were able to forage and defecate without the disorientation observed in larger structures. A critical improvement was the inclusion of a single honey frame on the western arena wall outside each colony. Bees foraged from this frame and disoriented bees left outside the colony clustered here overnight without starving to death. The arenas were enclosed in a single larger chamber (18.3 m W x 61.0 m L x 3.7 m H) covered with 40% shadecloth and cooled by an evaporative cooler. Daily temperature and humidity varied from 22°C to 40°C and 30% to 87% RH, a range comparable the monsoon season in Tucson (Tucson Climatological Monthly Reports, http://www.ncdc.noaa.gov/, National Climate Data Center, Asheville, NC, USA).

Eight colony constructs consisting of a queen, approximately 10,000 adult workers, and ten frames containing approximately equivalent proportions of capped brood, uncapped brood, honey, and empty cells, but no stored pollen, were assembled from donor field colonies. Each colony was installed in hive equipment consisting of a Langstroth deep box, a bottom board, an entrance reducer, a top-feeder, and a top cover. All colonies resumed brood rearing to some extent within 24 hours of setup. One day after the initial setup and pre-treatment overnight volatile collection (1d), colonies were either supplemented with pollen (pollen-supplemented) or completely deprived of pollen sources and stores (pollen-deprived) to simulate a brief pollen flow (n = 4 for each treatment). Pollen-supplemented colonies were provided with pollen frames with approximately 4,800 stored pollen cells, 250 g supplemental 9:1 pollen patty inside the colony, and ground corbicular pollen (~5 g/daily, same source as patty) on a steel baking pan outside the colony. Bees actively collected and stored the ground pollen. Pollen-deprived colonies were given an equivalent amount of honey frames to the pollen frames. Colonies were given water and allowed to feed outside *ad libetum* on dilute sucrose syrup solution (2:1 v/v) to mildly stimulate brood rearing. Brood and adults were periodically counted to assess the effect of pollen supplementation on colony population structure. Frame cell counts and adult worker number estimates were made the day after whole colony volatile samples were collected (1d, 7d, and 13d, see below for volatile collections). Eggs, younger larvae (L1 to L2), older larvae (L3 to L5) and capped brood were counted separately. Cell counts were made either visually or by examination of frame photographs. Because eggs and younger brood were difficult to see in photographs, all cell counts of eggs and younger larvae were verified by direct examination of the frames. Estimates of adult worker numbers were made by weighing the whole colony with and without the adult bees, and applying a bee fresh mass-to-individual bee conversion factor.

### Effects of pollen supplementation on young adult worker bee development

Nutritional conditions within each arena colony were estimated by examining the development of young adult workers in the colony environment. Newly emerged adult workers were obtained from field colonies by caging bees emerging from capped brood frames for 6-8 hours with a wire mesh cage. Fifty newly-emerged workers were paint-marked with Testor’s paint and carefully released into the top of each colony. Marked bees were sampled 12 days later into liquid nitrogen. Frozen bees were first dissected and weighed without the variable honey stomach contents to determine the fresh body mass (n = 4 colonies, 9 workers per colony). The growth of two tissues associated with nurse bee quality and worker maturation were estimated indirectly through their nutrient contents [65]. Freshly-dissected HPG were homogenized in 1000 μL PBS buffer by 100 μg 1.0 mm zirconium beads in a Bead Beater (BioSpec Products, Inc., Bartlesville, OK, USA) for 30 seconds (n = 4 colonies, 4 workers per colony). The resulting homogenate was centrifuged for 5 minutes and 25 μL of the supernatant was analyzed by a bicinchoninic acid (BCA) assay (Pierce BCA Protein Assay kit, Thermo Fisher Scientific, Waltham, MA, USA). Soluble protein contents were calculated by comparison against a standard curve generated by the albumin standard. Fat bodies were quantified by a chromic acid oxidation assay while attached to the dorsal abdominal cuticle (n = 4 colonies, 3 workers per colony) [65]. Fat body tissues were extracted in 1000 μL 2:1 chloroform: methanol solution, homogenized by 200 μg 1.0 mm zirconium beads in a Bead Beater (BioSpec Products, Inc., Bartlesville, OK) for 30 seconds, centrifuged for 10 minutes, and partitioned against 210 μL 0.25% KCl solution. Two hundred μL of the lower layer was dried down in a SpeedVac and incubated in 1.500 mL chromic acid in a sealed crimp glass vial at 95°C for 30 minutes. Fifty μL of the reacted solution was added to 100 μL concentrated sulfuric acid in a 96 well plate. Lipids were detected by the reduction of chromate (VI) to chromate (III) at 620 nm on a Gen-5 plate reader (Biotek, Inc., Winooski, VT, USA). Lipid contents were calculated by comparison of sample absorbance against a standard curve generated for oleic acid (Sigma, Saint Louis, MO, USA).

### Overnight collection of E-β-ocimene emissions from whole arena colonies

Volatiles were collected *in situ* from the airspace of whole intact arena colonies 0, 6, 9, and 12 nights after the start of pollen supplementation treatments [53]. Colonies were arranged such that the only significant openings were the entrance and a small gap in the top board. In 2011, validation checks confirmed that background stored pollen, honey, and hive materials other than the bees themselves did not release detectable ocimene levels. Volatiles were trapped from the colony headspace by a SuperQ 80/100 adsorbent filter (Hayes Separations, Bandera, TX, USA) attached in-line to an active vacuum pull system. We used ambient air drawn into the colonies rather than a controlled air source (compare with Carroll and Duehl, 2012 [53]) since outside air contained negligable ocimene. To sample air from the center of the colony, an elongated collection tube (30 cm long x 0.95 cm diameter rigid Teflon tube) was threaded through the front gap in the top board and positioned on the center topbar of the brood frames. A small cylinder of rigid Teflon tubing (0.32 cm diameter) was placed inside the end of the collection tube to keep bees from crawling inside. The other end was connected in-line to the adsorbent filter trap. The filter/collection tube apparatus was connected inline to a flowmeter-regulated (Aalborg, Inc., Orangeburg, NY, USA) vacuum source by Tygon tubing. Air was drawn through the filter at 650 mL/min by a vacuum pump. Volatiles were collected for 14 hours overnight (4:30 pm to 6:30 am) when colony bees were present.

### GC/MS analysis of E-β-ocimene emissions

Volatile analysis was performed by positive ion electron impact gas chromatography-mass spectrometry (EI GC-MS) on an HP 7890A gas chromatograph coupled to an HP 5975D mass spectrometer detector [53]. Exactly 1.0 µ L sample solution was injected at 220°C onto an Agilent HP-5MS column (30 m x 0.250 mm ID x 0.250 µm film; Agilent Technologies, Santa Clara, CA, USA) or an Agilent HP-1MS column (30 m x 0.250 mm ID x 0.25 µm film; Agilent Technologies, Santa Clara, CA, USA). Compounds were separated by oven temperatures programmed from 40°C (0.5 min hold) to 220°C at 15°C/min and held for 12 min. Samples were injected in a 1:10 split mode and helium was used as a carrier gas at a flow rate of 1.2 mL/min. Sample compounds were identified by comparison of retention times and mass spectra with authentic standards (Sigma, St. Louis, MO, USA) and mass spectra libraries (NIST and Department of Chemical Ecology, Göteburg University, Sweden). Volatile emission rates for whole colony samples were calculated by comparing the E-β-ocimene peak area with authentic standards and correcting for processing error against the peak area of the nonyl acetate internal standard. Ocimene emissions were kept on a relative scale (ocimene peak area units released per hour) because the whole colony collections may not have captured all ocimene volatiles released by the colony. For each colony, all whole colony emission rates were scaled against overnight emissions on the night before feeding treatments began (0d =  100%).

### Effects of ocimene supplementation on brood care and brood cannibalism

A second experiment was conducted to determine if ocimene emissions associated with pollen supplemented increased egg retention in summer dearth colonies. This trial was conducted near the end of a summer dearth when brood rearing had been limited and erratic for one month. Each nuc was converted to a broodless microcolony containing a queen, approximately 3,000 adult workers, a partially filled honey frame with at least 30% open cells and an empty cell frame devoid of brood and stored food. All brood and excess adult workers were removed and isolated in storage to avoid worker return to the colony. The remaining workers formed a nest center that covered the entirety of the space between the two adjacent frames. Half of the nucs were provisioned with 6 capillary tube release vials embedded in the empty cell frame on the side facing the honey frame (S2 Fig, after volatile release method of D’Alessandro et al., 2006 for wasps [64]). Each vial consisted of a 2.0 cm length section of 100 µL capillary tube (VWR catalog 53432-921, Radnor, PA, USA) embedded into a 2.0 mL red rubber crimp cap vial containing 200 µ L E-β-ocimene (Sigma, St. Louis, MO, USA). Vials were embedded 3 cm from the frame edge within the nest perimeter. A constant ocimene release rate was set from the saturated vial interior through the tube to the outside environment by the tube length, tube diameter, and relatively even temperature maintained by surrounding nest workers. Each capillary tube release vial emitted approximately 26 µg E-β-ocimene/hr, or the approximate equivalent of about 624 L2-L3 larvae, for a total emissions equivalent of 157 µg E-β-ocimene/hr, or the equivalent of 3,744 L2-L3 larvae per colony (after Maisonnasse et al., 2010 [48]).

Each nuc was isolated for 96 hours (development time to 1^st^ instar larvae) to allow for queen oviposition and worker brood care. At the endpoint, the total number of nuc workers were estimated from frame coverage and the frames examined for eggs and larvae. Representative nest workers were sampled from each colony on dry ice for examination of HPG and fat bodies. The cells were also closely examined for the presence of mass provisioned larval jelly, a sign of worker support for brood rearing. Detection of eggs, young larvae, and larval jelly was assisted by blacklight illumination of the frames since these materials faintly fluoresce under UVA (395 nm) LED flood lights (Waveform Lighting, Vancouver, WA, USA; S3 Fig). Frames were temporarily stored in a 4°C cooler to stop egg and brood development before full inspection.

### Statistical analysis

All statistics were performed using SAS 9.4 (SAS, Inc., Cary, NC, USA). Data sets were checked for normality by Shapiro-Wilks tests and examination of residuals (PROC UNIVARIATE). For the first experiment, whole colony ocimene emissions and the number of immature bees in major developmental subpopulations were compared across days since supplementation by a repeated measures one-way ANOVA (PROC MIXED DATA with REPEATED). Worker fresh masses were compared across pollen supplementation treatments by a two sample t-test (PROC TTEST). Soluble protein contents of hypopharyngeal glands and abdominal (fat bodies) lipid contents from 12 day-old worker bees were compared across pollen supplementation treatments by a non-parametric Wilcoxon rank sum test (PROC NPAR1WAY). For the second experiment, the total number of eggs and 1^st^ instar larvae found in each nucleus colony were compared across ocimene treatments by a Wilcoxon rank sum test (PROC NPAR1WAY).

## Results

### Effects of pollen supplementation on worker subpopulations

**Fig 1:**
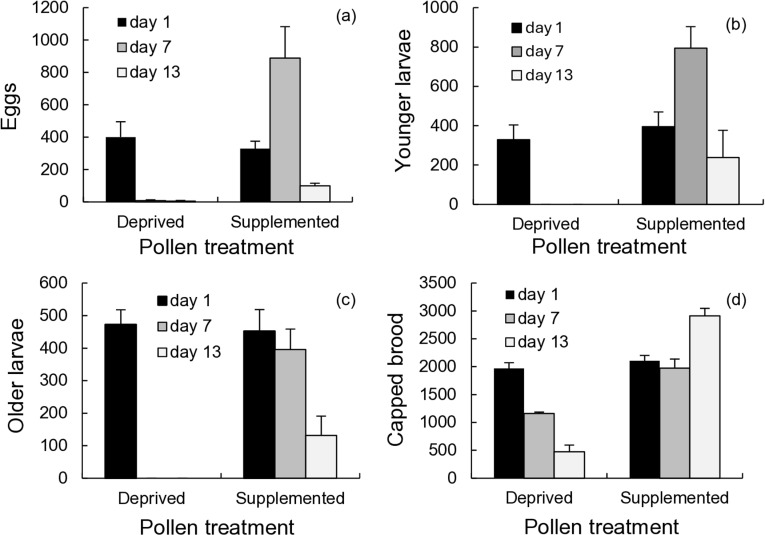
Average number (means ±  SE) of (a) eggs, (b) younger larvae, (c) older larvae, and (d) capped brood in pollen-supplemented and pollen-deprived flight arena colonies (n = 4 colonies). The number of bees present in each age cohort were counted from each colony the day after the overnight volatile collections. Days refer to the number of days that have elapsed since the beginning of forage treatments.

Pollen-supplemented colonies responded to pollen influx by rearing more brood (Fig 1a–d; repeated measures one-way ANOVA, F = 89.67, df = 1, p < 0.001 (capped brood); F = 9.53, df = 1, p = 0.023 (older larvae); F = 18.96, df = 1, p = 0.005 (younger larvae); F = 16.54, df = 1, p = 0.007(eggs)). Brood numbers increased first by day 7 among eggs and younger larvae that contribute significantly to ocimene emissions, followed by capped brood by day 13. By day 13, the period of enhanced brood rearing had ended as egg and younger larvae numbers declined below levels observed at the beginning. In contrast, brood rearing sharply declined in pollen-deprived colonies, eventually depleting these colonies of most of their brood complement. Eggs, younger brood, and older brood rearing almost completely ceased by day 7, while capped brood declined considerably by day 13. Older larvae appear not to have been cannibalized but were simply not replaced.

### Effects of pollen supplementation on whole colony E-β-ocimene emissions

**Fig 2:**
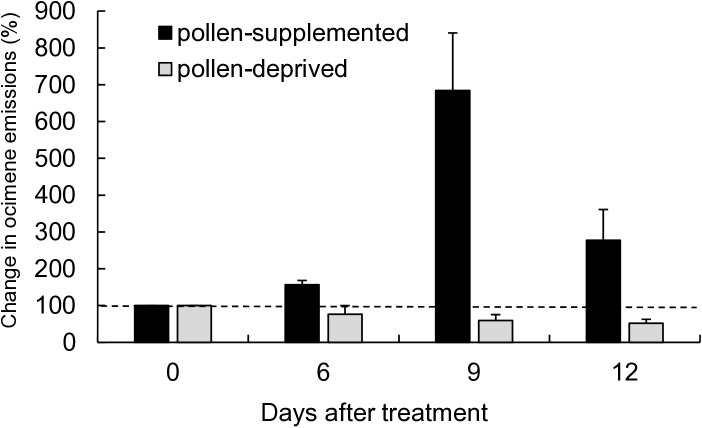
Relative E-β-ocimene emissions (means ±  SE) of pollen-supplemented and pollen-deprived flight arena colonies (n = 4 colonies). For each colony, all whole colony emission rates were scaled to its overnight emissions on the night before feeding treatments began (0d =  100%, represented by the dotted line).

Increased brood rearing in pollen-supplemented colonies coincided with significantly higher whole colony ocimene emissions 6, 9, and 12 nights after initial pollen feeding. Overnight ocimene emissions from arena colonies were strongly affected by pollen supplementation treatment (Fig 2; repeated measures one-way ANOVA, df = 1, F = 85.11, p < 0.001 (treatment); df = 3, F = 3.30, p = 0.0443 (timepoints); df = 3, F = 7.97, p = 0.0014 (treatment*timepoint)). Bees in the pollen-supplemented treatment responded to the pollen influx with increased ocimene production. Ocimene emissions of pollen-supplemented colonies increased to 685% of original emissions by night 9, then decreased to 277% on night 12. Notably, both elevated brood rearing and ocimene emissions continued in pollen-supplemented colonies for days after feeding. By contrast, pollen-deprived colonies experienced declines in ocimene emissions consistent with reductions in ocimene producing brood subpopulations. Ocimene emissions declined by 24% in pollen-deprived colonies between night 0 and night 6 and decreased further to 59% and 52% of original emissions by night 9 and night 12. Background ocimene emissions in colonies lacking eggs or young brood could likely be attributed to other ocimene producers such as the queen, capped brood, and the workers themselves.

### Effects of pollen supplementation on adult worker development

Physiological comparisons 12d old adult workers reared in treatment arena colonies demonstrated that pollen supplementation substantially improved worker quality (S4 Fig a–c). Fresh masses of 12d old adult workers in pollen-supplemented colonies did not differ from workers in pollen-deprived colonies (two sample t-test, df = 70, t = 1.44, p = 0.1540). However, HPG sizes (as indicated by soluble protein content) were significantly higher in bees from pollen-supplemented colonies than pollen-deprived colonies (Wilcoxon rank sum test, Z = 3.892, p < 0.0001). Similarly, abdominal lipid contents (fat bodies) were higher in bees from pollen-supplemented colonies than pollen-deprived ones (Wilcoxon rank sum test, Z = 3.3775, p = 0.0007).

### Effects of ocimene supplementation on egg and young larvae retention

Pre-experiment validation checks found the 2024 summer dearth nucs to be only moderately nutritionally stressed as opposed to the 2011 drought colonies. All queens were observed ovipositing daily despite the dearth and all nucs reared small, if erratic, numbers of brood during the three weeks before the trial. Nest workers in all nucs had fully developed HPG and fat bodies. Nucs from both treatments had similar adult worker populations both at the beginning and end of the experiment and did not gain or lose appreciable numbers of workers due to drift or treatment attraction.

**Fig 3:**
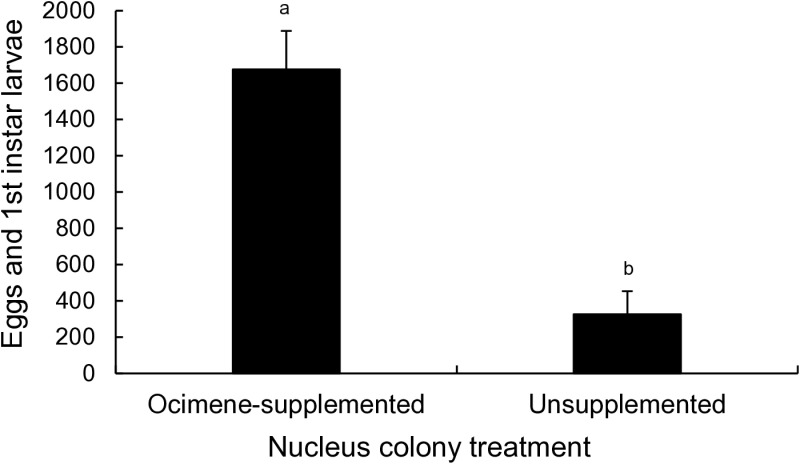
Average total number of eggs and first instar larvae (means ±  SE) observed in E-β-ocimene-supplemented and unsupplemented nucleus colonies (n = 12 colonies). Eggs and first instar larvae were counted 4 days after treatment initiation. Means that do not share a superscript differ by a Wilcoxon rank sum test (p < 0.05).

Synthetic ocimene supplementation substantially improved egg and L1 larvae retention in initially broodless dearth colonies (Fig 3; Wilcoxon rank sum test, Z = 0.8942, p = 0.0162). All ocimene-supplemented colonies retained some eggs and did not completely cannibalize eggs and L1 larvae. Ocimene supplemented colonies had on average 1,676 eggs and first instar larvae after four days. By contrast, 50% of unsupplemented colonies contained no eggs or larvae – evidence of concerted brood cannibalism. On average, unsupplemented colonies had 326 eggs and first instar larvae. Young brood cells mass provisioned with larval jelly were overwhelmingly observed in ocimene supplemented colonies (84% of total cells) rather than unsupplemented colonies (16% of total).

## Discussion

Brood regulating mechanisms in social insect colonies change based on sensitivity to critical factors such as brood presence, forage and food availability, and nutritional stress. In this experiment, we observed that pollen supplementation that stimulated brood rearing also increased ocimene signaling. Brood rearing in pollen-supplemented colonies expanded over several days, a result consistent with known effects of pollen availability on brood rearing in nutritionally-stressed colonies [66,67]. The elevation in colony ocimene emissions observed in pollen-supplemented colonies likely reflected increases in the subpopulations of ocimene-producing brood. Young larvae and eggs have both been identified as producers of ocimene in honey bee colonies, although unusually for colony regulating pheromones, other castes and life stages are minor producers as well [48,52,53,55,56]. These results are in agreement with current concepts of colony brood pheromone signaling – that pheromone signaling by brood largely reflects the subpopulation size of pheromone producers [48,63].

The increase in brood rearing among pollen-supplemented colonies was likely due in part to improved nutritional conditions within the colony as demonstrated by young worker development. Worker nursing capabilities depend largely on food availability during young adult development, which varies considerably. Workers reared in pollen-supplemented colonies were not larger than workers from pollen-deprived colonies but had larger tissues critical for nursing and internal nutrient stores [9,38]. During the 2011 drought, bees from both treatments were reared as larvae by severely malnourished workers, a legacy that likely impacted their size. However, both HPG and fat bodies primarily grow during young adult development when newly emerged workers depend on available pollen resources and trophallactic interactions for food [36,37]. Alternatively, pollen-deprived adult workers may have matured faster in nutrient poor environments and experienced atrophy of HPG and fat bodies associated with precocious foraging [34,35].

Colony nutrition impacts not only adult worker quality, but also the brood rearing activities of workers required to sustain the colony. Synthetic ocimene releases simulating young brood presence were sufficient to increase egg retention in broodless summer dearth colonies. One unusual feature was the relatively modest amounts of ocimene required to increase egg retention in dearth colonies. The amount of synthetic ocimene used here represented the emissions from 3,744 L2-L3 larvae, a number outside the daily oviposition capacities of queens but biologically meaningful for a colony with brood [48,68]. An important consideration is that ocimene by itself might not increase egg retention alone but rather may prime worker sensitivity to forage cues. Bees in both experiments were exposed to dilute sugar solutions (2:1 dilute sucrose syrup in 2011, small amounts of tamarisk honey in 2024). These inputs either signal or simulate incoming nectar throughout the colony, a known stimulant of brood rearing in nutritionally-stressed colonies [25]. Periodic feeding with dilute sucrose syrup was necessary to sustain brood rearing during the severe drought the 2011 colonies experienced. Our experiment also involved the complete removal of small numbers of brood from nucs before ocimene treatment, a necessary artifact that likely impacted workers. Brood presence has been shown to stabilize retention of other life stages and the colony identity itself. Queen retention during package installation (a simulated swarm) is much higher when brood is included in the new colony [69]. Additional studies will be needed to determine if ocimene stimulates brood rearing on its own or primes workers to accompanying forage or brood cues. Ocimene treatment effects on egg and brood numbers were not likely due to queen oviposition alone given known responses of queens to poor nutrition. Queens tend to continuously lay eggs in all but the most pronounced dearths and do not rapidly respond to shifts in colony nutrition [18,70]. Queens are known to alter their oviposition in response to poor colony nutrition by laying larger eggs [71]. While we did not measure oviposition directly, queens in all nucs were observed actively ovipositing during each day of the ocimene supplement trials.

Our estimates of ocimene emissions from young L2-L3 larvae used in the ocimene supplementation trial originated from Maisonnasse et al 2010 and were substantially higher than those calculated by Carroll and Duehl, 2012 [48,53]. In our previous study, we employed a method of volatile sampling (push-pull air-vacuum collection system on HayeSep Q 80/100 absorbent filters) that collected all emitted ocimene from relatively undisturbed larvae on brood frames [53]. Other studies, such as Maissonnasse and coauthors (2009; 2010), captured volatiles from isolated larvae with a SPME fiber, while more recent studies by Noёl and coauthors (2023) have used SPME to sample intact brood [48,52,56]. SPME collections are known to be more sensitive than absorbent filter collections but more difficult to calibrate for quantification and are therefore often compared on a relative scale in compound peak area units (see SPME guidelines Journal of Chemical Ecology; Noёl et al., 2023 [52]). A more important consideration is the context of synthetic ocimene releases attempted in these studies. Most previous studies examined the effects of ocimene signaling simulating releases from large to very large numbers of brood on foraging behavior, worker nursing and reproductive physiology [48,56–59,63]. As such, synthetic ocimene releases designed to simulate large young larvae subpopulations were appropriate. By contrast, our study replicated ocimene releases from a moderate number of young larvae (3,744 L2-L3), such as one would find in dearth colonies with modest brood populations. Notably, our ocimene supplementation experiment did not deploy synthetic releases at rates similar to near broodless dearth colonies. Further studies need to be conducted to test ocimene’s effects on severely impacted dearth colonies (i.e., when broodless colonies resume brood rearing).

Small amounts of ocimene functioning as a cue to resume brood rearing may be particularly effective in dearth-stressed colonies. Resumption of brood rearing after extensive brood cannibalism in nutritionally-stressed colonies invariably start with retention of a small number of eggs and young larvae. Ocimene is a reliable signal of the presence of young brood and a viable egg-laying queen [48,54,55]. At the level of individual brood, ocimene is also known to increase worker visits to both younger and older larvae under nutritionally stressed situations [60]. Ocimene may also signal the presence of quality brood worth expending limited nursing resources on. Larval rearing is known to impose considerable costs on nursing workers both in terms of internal nutrients and reduced worker lifespans [31,45]. Weakened colonies are highly susceptible to worker depletion and substandard worker quality especially at the transition between extended dearth and new forage availability [72–74]. Ocimene may also signal workers to engage in pollen foraging near the end of a dearth when forage starts to become more abundant. Higher ocimene emissions near a dearth’s end may also accelerate worker maturation to foragers at a time when forage become more available, brood populations are small, and contradicting brood ester pheromone (BEP, which slows adult worker maturation) signaling from older brood may be reduced [48].

Our study focused narrowly on ocimene effects on egg and L1 larvae removal because those stages were the main immatures that were being removed from our summer dearth colonies. We avoided additional assessments through later stages because repeated colony disturbance increases brood cannibalism artifacts. Ocimene increased egg retention over the short term but ocimene’s impact on larval stages most directly tended by workers remains unknown. Further studies are needed to determine if ocimene reduces brood cannibalism through all of larval development. Young to middle aged larvae (L1-L3) have been reported as the main targets of brood cannibalism [16,18]. Schmickl and coauthors (2003) noted that workers first sharply reduce care of younger larvae but tend to cannibalize brood through middle aged larvae, focusing preferentially on neglected younger larvae [18]. Younger L2 larvae are much less resistant to starvation than older L4 larvae and begin to die within hours of worker neglect [60]. Similarly, the number of younger larvae, but not older larvae, workers are willing to rear depends on pollen availability in the colony [17]. Indications from our experiment suggest that ocimene increases mass provisioning of L1 larvae, the first significant nutritional input from workers to brood [9,11]. Almost all L1 brood cells with visible larval jelly (84%) in our study were in ocimene supplemented nucs.

One final consideration not addressed by our experiments is how ocimene signaling from individual bees changes under nutritional stress. We did not quantify ocimene emissions among pollen supplemented bees at the level of single developmental stages. The increased ocimene emissions observed in our pollen-supplemented arena colonies were sampled from whole colonies containing many different ages and castes of bees. Earlier researchers have found that younger L2 and older L4 larvae increase ocimene emissions when starved (left unattended by workers) up to 6 hours [60]. However, the extent of such hunger volatiles in whole colonies experiencing chronic pollen deprivation with nest workers present is less clear. The production of other colony pheromones has been shown to change in response to stressors. Queen-produced queen mandibular pheromone (QMP) and forager-produced ethyl oleate (EO) vary seasonally, while QMP release rates are altered in *Nosema ceranae* infected queens [65,75,76]. Such findings suggest that pheromone communication should be considered in context, namely, sensitive to stressors and interpreted by pheromone receivers that adapt colonies to changes in colony conditions.

## Supporting information

S1 Fig Small flight arena used for pollen-supplementation experiment. Each arena was partioned into four sections (each containing a colony) by fine nylon mesh.
(TIF)

S2 Fig Placement of six capillary tube release vials used to supplement colonies with synthetic ocimene volatiles. Each vial was embedded into wax comb approximately 3 cm from the frame periphery. Small amount of tamarisk nectar are visible as dark cells. Partially oxidized synthetic ocimene is yellow in the vials.
(TIF)

S3 Fig Visualization of eggs, young larvae, larval jelly, stored pollen, honey, and other brood cell components in a brood frame by blacklight (UVA 395 nm) illumination. Many frame materials fluoresced under the UVA light.
(TIF)

S4 Fig (a–c) Young adult worker development in pollen-supplemented and pollen-deprived colonies. (a) Fresh weight masses (means ±  SE), (b) soluble protein contents of hypopharyngeal glands (means ±  SE), and (c) abdominal lipid contents (including fat bodies, means ±  SE) of 12 day-old adult workers reared in pollen-supplemented and pollen-deprived flight arena colonies (n = 4 colonies, 9 (mass), 4 (HPG protein contents), and 3 (abdominal lipid contents) workers per colony). Highly variable honey stomach contents were removed before the fresh body mass was determined. Means that do not share a superscript differ by a two-sample t-test (mass) or a Wilcoxon rank sum test (HPG soluble protein and abdominal lipid contents; p < 0.05).
(TIF)

S5 File Data used in experiment. (XLSX)
