# Supplementary figures and images for: E-B-ocimene and brood cannibalism: Interplay between a honey bee larval pheromone and brood regulation in summer dearth colonies
Source: PLoS One. 2025 Feb 6;20(2):e0317668. doi: 10.1371/journal.pone.0317668 (PMC11801708; doi:10.1371/journal.pone.0317668)

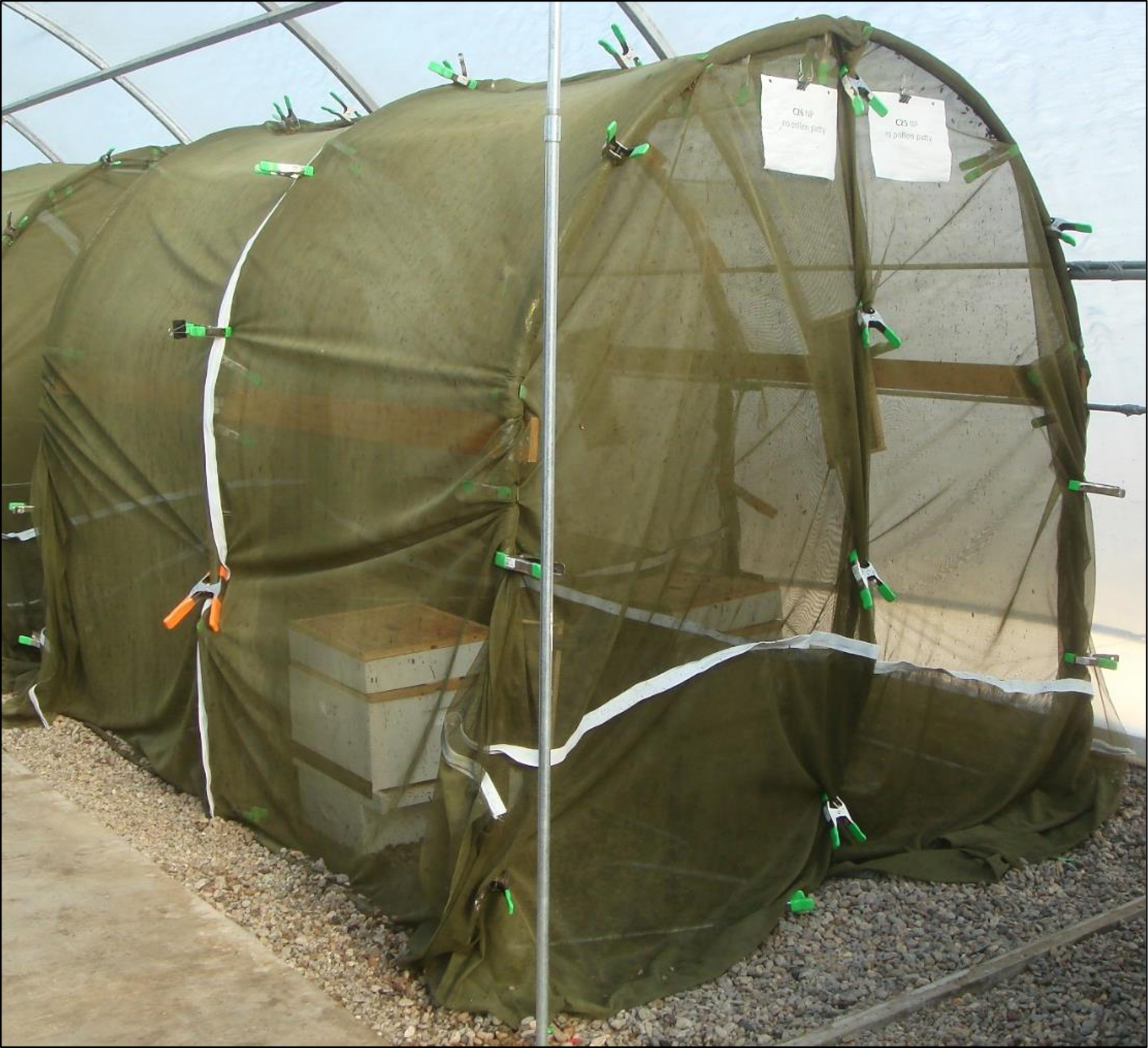

Supplement: S1 Fig — Each arena was partioned into four sections (each containing a colony) by fine nylon mesh. (TIF) [file pone.0317668.s001.tif]

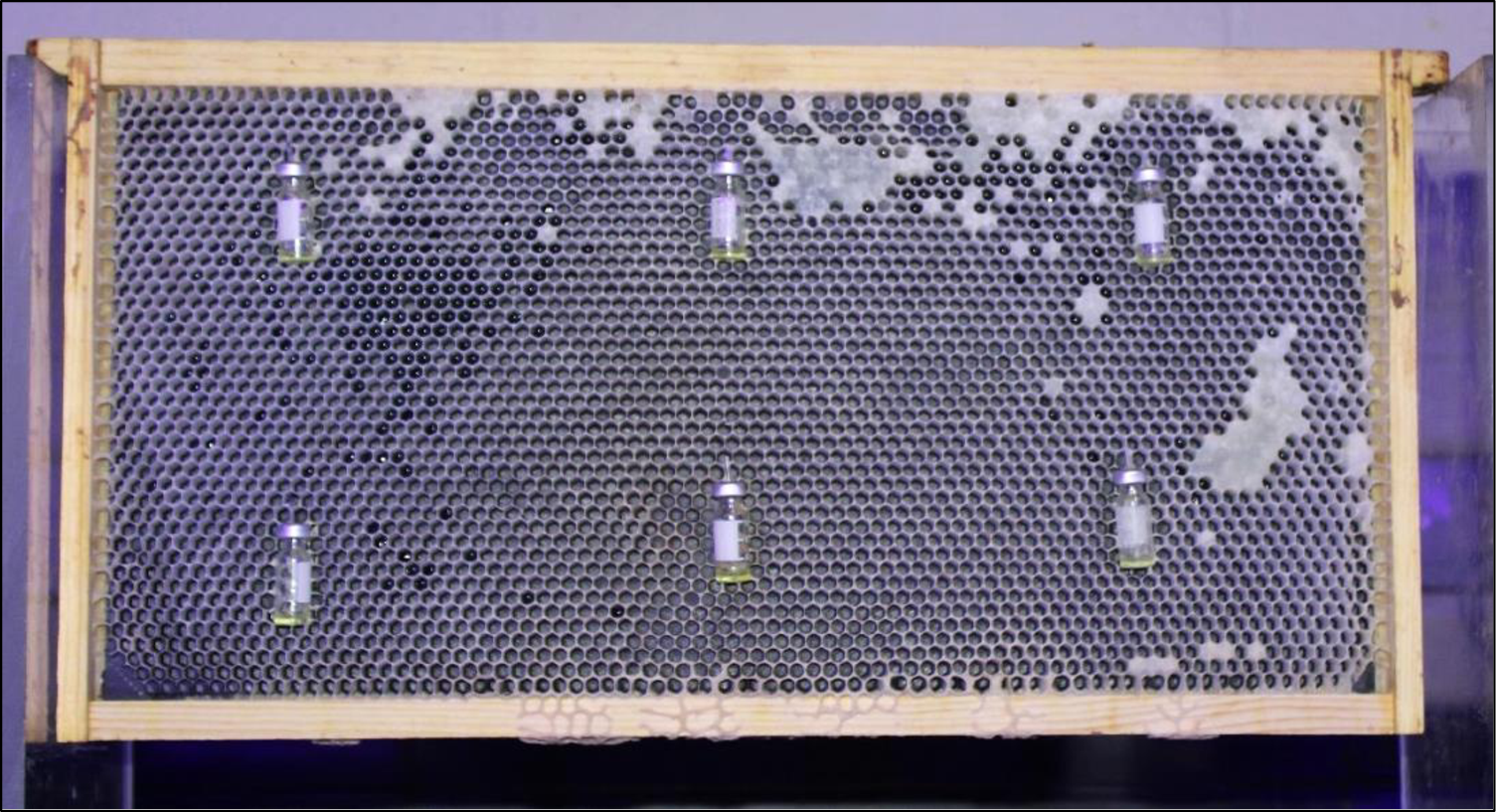

Supplement: S2 Fig — Each vial was embedded into wax comb approximately 3 cm from the frame periphery. Small amount of tamarisk nectar are visible as dark cells. Partially oxidized synthetic ocimene is yellow in the vials. (TIF) [file pone.0317668.s002.tif]

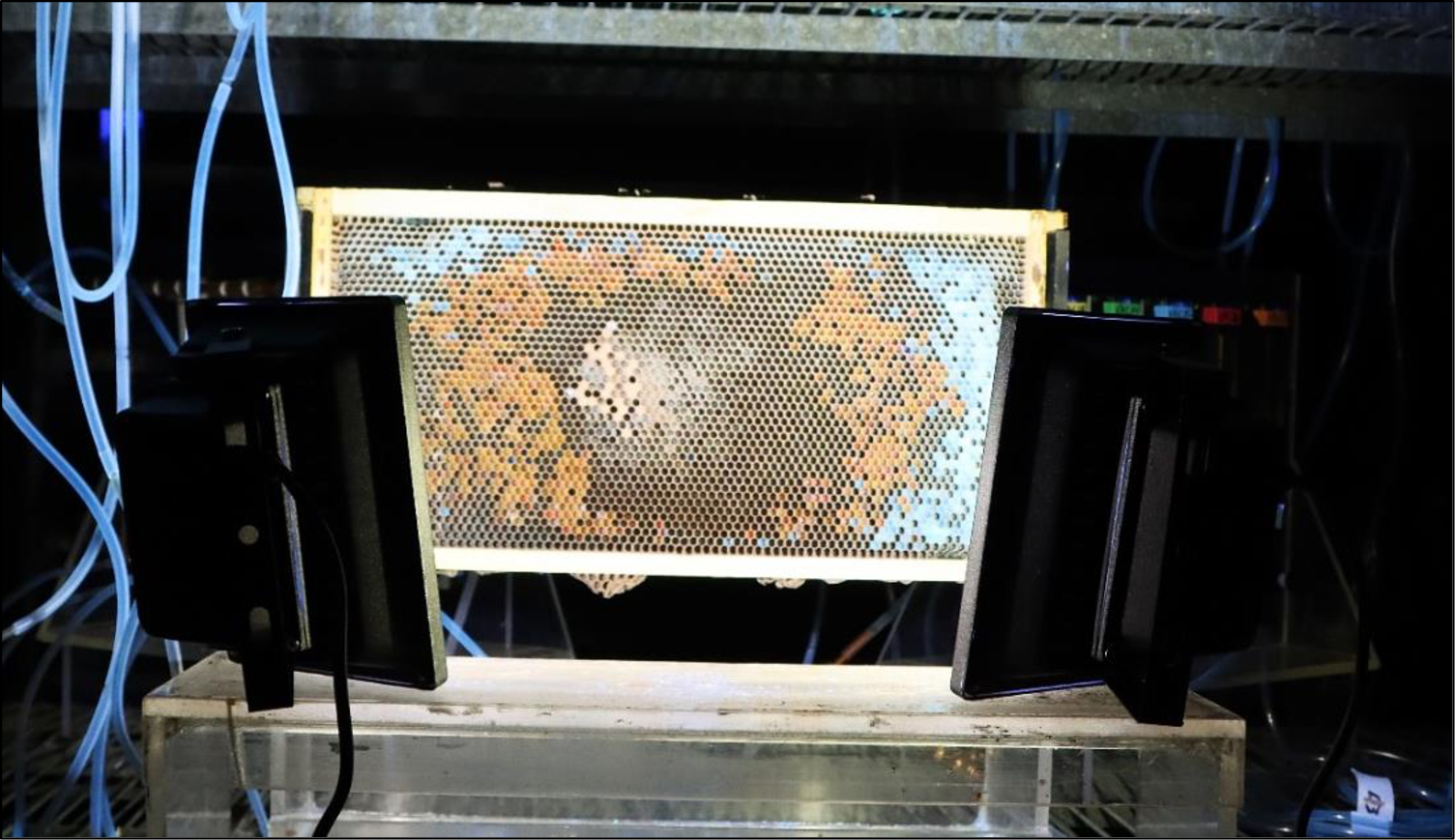

Supplement: S3 Fig — Many frame materials fluoresced under the UVA light. (TIF) [file pone.0317668.s003.tif]

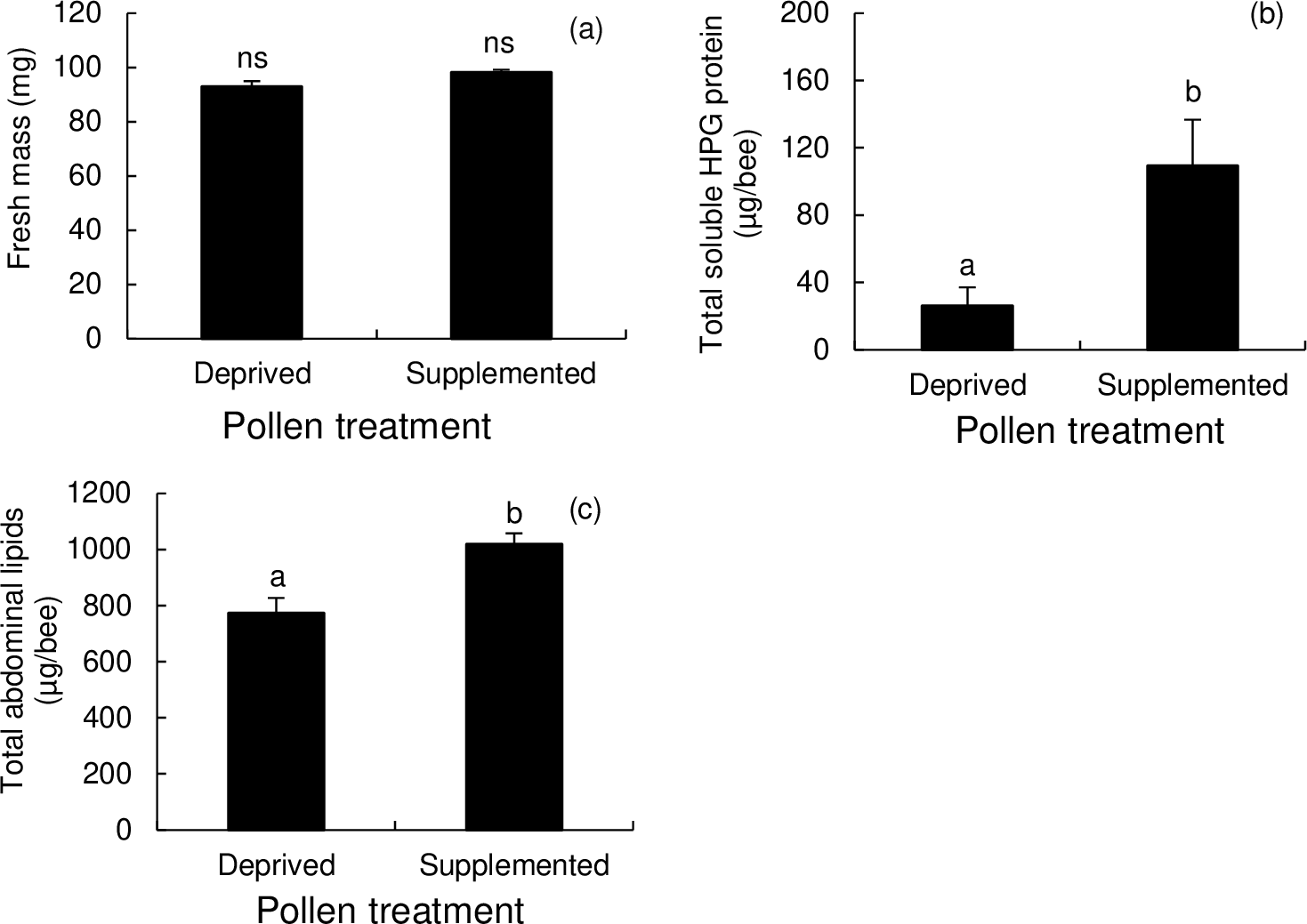

Supplement: S4 Fig — (a) Fresh weight masses (means ± SE), (b) soluble protein contents of hypopharyngeal glands (means ± SE), and (c) abdominal lipid contents (including fat bodies, means ± SE) of 12 day-old adult workers reared in pollen-supplemented and pollen-deprived flight arena colonies (n = 4 colonies, 9 (mass), 4 (HPG protein contents), and 3 (abdominal lipid contents) workers per colony). Highly variable honey stomach contents were removed before the fresh body mass was determined. Means that do not share a superscript differ by a two-sample t-test (mass) or a Wilcoxon rank sum test (HPG soluble protein and abdominal lipid contents; p < 0.05). (TIF) [file pone.0317668.s004.tif]
